# Comparative Assessment of Point Shear Wave Elastography (pSWE) and Two-Dimensional Shear Wave Elastography (2D-SWE) in the Diagnostic Evaluation of Simple Liver Cysts and Liver Hemangiomas

**DOI:** 10.3390/medicina61111940

**Published:** 2025-10-29

**Authors:** Emiliya Lyubomirova Nacheva-Georgieva, Daniel Ilianov Doykov, Bozhidar Krasimirov Hristov, Desislav Ivanov Stanchev, Iliya Stoyanov Todorov, Zhivko Georgiev Georgiev, Katya Angelova Doykova, Siyana Emilova Valova, Krasimir Iliev Kraev, Petar Angelov Uchikov

**Affiliations:** 1 Second Department of Internal Diseases, Section of Gastroenterology, Medical University of Plovdiv, 4000 Plovdiv, Bulgaria; daniel_doykov@abv.bg (D.I.D.); hristov.bozhidar@abv.bg (B.K.H.); dessislavs@gmail.com (D.I.S.); iliatodorov014@gmail.com (I.S.T.); 2 Clinic of Gastroenterology, University Hospital Kaspela-Plovdiv, 4001 Plovdiv, Bulgaria; 3 Department of Prosthetic Dentistry, Medical University Plovdiv, 4000 Plovdiv, Bulgaria; jivko_169@abv.bg; 4 Department of Radiology, Medical University of Plovdiv, 4000 Plovdiv, Bulgaria; katya.doykova@mu-plovdiv.bg; 5 Second Department of Internal Diseases, Section “Nephrology”, Medical Faculty, Medical University of Plovdiv, 4002 Plovdiv, Bulgaria; siyanavalova@abv.bg; 6 Department of Propedeutics of Internal Diseases, Medical Faculty, Medical University of Plovdiv, 4002 Plovdiv, Bulgaria; kkraev89@gmail.com; 7 Department of Special Surgery, Faculty of Medicine, Medical University of Plovdiv, 4002 Plovdiv, Bulgaria; puchikov@yahoo.com

**Keywords:** simple liver cyst, liver hemangiomas, pSWE, 2D-SWE

## Abstract

*Background and Objectives*: What led to the development of elastography was the emerging need for a method that could objectively and accurately assess the stiffness of internal structures. As a result, a distinction between normal from pathological tissues becomes possible. Objective: To evaluate non-invasive elastographic techniques, point shear wave elastography (pSWE) and two-dimensional shear wave elastography (2D-SWE), as methods for differentiating simple liver cysts from liver hemangiomas. *Materials and Methods*—a total of 63 patients—32 with simple liver cysts and 31 with liver hemangiomas were analyzed. The purpose was to determine the values of the average trend (arithmetic mean or median according to the data distribution) as well as the reference intervals of SWV for both methods in the above-mentioned patients. Final diagnoses were confirmed by contrast-enhanced computed tomography (CECT). *Results*: The pSWE SWV values (median) for simple hepatic cysts showed an average trend of 1.14 m/s, with an upper limit of 3.33 m/s and a lower limit of 0.35 m/s. For 2D-SWE, the average trend for simple hepatic cysts was 1.00 m/s, with an upper limit of 1.54 m/s and a lower limit of 0.65 m/s. For liver hemangiomas, the average trend in pSWE was 1.36 m/s, with an upper limit of 3.22 m/s and a lower limit of 0.57 m/s. For 2D-SWE, the average trend was 1.34 m/s, with an upper limit of 2.27 m/s and a lower limit of 0.80 m/s. Findings in our work mainly serve as reference values. *Conclusions*: The accurate diagnosis of liver diseases is of paramount importance when it comes to the approach and treatment of individual benign liver lesions. Early diagnosis of focal liver lesions remains a challenging task.

## 1. Introduction

Elastography is an ultrasound-based technique developed in the 1990s for measuring the stiffness of tissues and organs [1,2,3]. Increased tissue stiffness is a fundamental feature of pathological changes, making elastography a valuable tool for clinical diagnostics. The method is applicable in various fields of medicine, and there are many different elastography modalities such as transient elastography, strain imaging, endoscopic ultrasound elastography, MR elastography, and shear wave imaging (SWE). The latter includes point shear wave elastography (pSWE) and two-dimensional shear wave elastography (2D-SWE) [4,5,6,7]. Elastography’s significance has been further validated since its modalities are used in the diagnosis of diffuse and focal liver lesions [8,9,10], breast diseases [11,12], thyroid pathology [13], pancreatic diseases [14,15], prostate gland [16], lymph nodes [17], intestinal diseases [18], and diseases of the musculoskeletal system [19].

In gastroenterology, the ability to differentiate focal liver lesions and determine appropriate therapeutic strategies is of particular importance. Due to its non-invasive nature, repeatability, wide availability, ease of use, and low cost, elastography has gained a widespread clinical application. In many countries, it is now a routine diagnostic tool for assessing liver stiffness and staging fibrosis in patients with chronic liver disease [20,21]. Its clinical utility has also been supported by numerous studies [22,23,24].

Conventional ultrasonography offers crucial advantages such as low cost, real-time imaging, and a lack of radiation exposure. However, information on tissue mechanical properties is not provided. While both computed tomography (CT) and magnetic resonance imaging (MRI) are highly sensitive, their relatively higher cost can be seen as a disadvantage. An additional drawback of CT scans is the ionizing radiation. On the other hand, the use of MRI is further limited by a contraindication in patients with claustrophobia, cardiac pacemakers, implanted cardioverter-defibrillator, and metallic implants.

Regardless of the promising results, some restrictions were observed. As our study demonstrates, pSWE and 2D-SWE should not be considered as a substitute for benign focal liver lesion CECT because the stiffness values overlap.

Further research and work in this direction are required in order to validate the reference intervals and make elastography a suitable method for differentiating simple liver cysts from liver hemangiomas.

## 2. Objective

To evaluate non-invasive elastographic techniques—pSWE and 2D-SWE—as methods for differentiating simple liver cysts from liver hemangiomas.

## 3. Materials and Methods

Between 2019 and 2025, a total of 63 patients were enrolled at the Gastroenterology Clinic of the University Hospital “Kaspela” in Plovdiv. The following patient groups were included:

### 3.1. Patients with Liver Hemangiomas-31

All patients were ≥18 years of age and provided their written informed consent prior to inclusion, in accordance with the ethical standards of the Declaration of Helsinki and good clinical practice guidelines.

Data were collected on the patients’ age and gender, BMI, presence of risk factors such as alcohol consumption and smoking; values of ASAT, ALAT, GGT, ALP, total bilirubin, direct bilirubin, AFP, presence of liver cirrhosis, presence of ascites, transverse and longitudinal lesion size. The stiffness of simple liver cysts and liver hemangiomas were determined by pSWE and 2D-SWE integrated using the same ultrasound machine, Esaote MyLab^TM^ 9 Exp; Esaote, Genova, Italy. A convex C1-8IQ probe transducer was used. Ten elastographic measurements were performed with a validation criterion-median interquartile range (IQR/M) < 30. All patients fasted for at least a minimum of two hours and rested for 10 min prior to the examination to avoid falsely elevated liver stiffness measurements. The measurement box for both methods was placed parallel to the liver capsule. The upper edge of the measurement box should be placed 1.5 to 2.0 cm apart from the liver capsule to minimize the effect of reflection artifact.

This section presents the results of the statistical analysis of data on the ability of non-invasive elastographic methods, pSWE and 2D-SWE, to differentiate simple liver cysts from liver hemangiomas. It is organized by order of the scientific tasks: first, to investigate the methods pSWE and 2D-SWE for the diagnosis of the above-mentioned patient groups and second, to determine the values of the average trend (arithmetic mean or median according to the data distribution) and the reference intervals of SWV for pSWE and 2D-SWE in the same patients.

The inclusion criteria were as follows: patients of age (>18 years) with simple liver cyst or liver hemangioma clearly visualized at gray scale ultrasound; localized at least 2 cm under Glisson’s capsule with a maximum depth of 7.5 cm.

Exclusion criteria for our research were: age < 18 years; liver lesions different than simple liver lesion or liver hemangioma, complex cysts, lesions less than 2 cm. Deep-seated lesions localized in the left liver lobe were also avoided, since this area is close to the heart and is subjected to artificial motions due to heart pulsations.

Following the ethical recommendations of the Helsinki Declaration and Good Clinical Practice, all patients signed their informed consent before entering the study.

### 3.2. Statistical Analysis

Demographic and clinical data for patients with simple liver cysts and liver hemangiomas were presented using descriptive statistics. Binary variables (gender, alcohol consumption, smoking) were presented as numbers and percentages (%), and gender-based comparisons were made using Fisher’s exact test. Continuous variables (age, lesion size, laboratory parameters) with normal distribution were represented by both the mean and standard deviation (SD), and an independent samples *t*-test was used to compare the male and female patients. Continuous values that did not follow a normal distribution were presented as the median and interquartile range (IQR). The Mann–Whitney U test was performed to compare the male and female patients. Three of the variables (pSWE SWV m/s, pSWE depth of region of interest in mm, and 2D-SWE SWV m/s) showed a lack of normal distribution, and the average trend was represented by the median and interquartile range (IQR). Values in both genders were compared using the Mann–Whitney U test.

The fourth variable (2D-SWE depth in mm) showed a normal distribution, and the average trend was expressed by the arithmetic mean and standard deviation (SD). Genders were compared by the independent-samples *t*-test. Before determining the SWV reference values for pSWE and 2D-SWE, the data were transformed using a logarithmic function [x’ = log10(x), where x = the original dimension for each patient] to achieve a normal distribution. Then, a reverse transformation was performed to the original measurement scale. The reference intervals were determined using the Robust method, which is recommended for samples of less than 100 cases.

Appropriate graphs, including pie charts, box plots, and reference interval plots, are included to illustrate the more important trends.

## 4. Results

### 4.1. Information on Patients with Simple Liver Cysts

#### 4.1.1. Basic Information on Patients with Simple Liver Cysts

**Figure 1:**
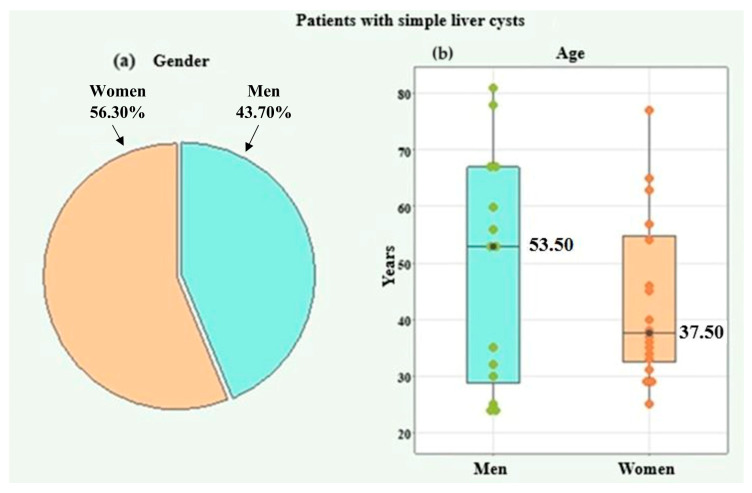
Distribution by gender (**a**) and age (**b**) of patients with simple liver cysts.

The first study group consisted of 32 patients with simple liver cysts. The gender distribution included 14 (43.70%) males and 18 (56.30%) females (Figure 1a). Due to the lack of normal distribution according to the Shapiro–Wilk test (*p* = 0.002), the age of the patients was represented by the median and interquartile range (IQR). For the entire group, the age range was 24 to 81 years, with a median of 39 years (IQR = 28). For men, the age range was 24 to 81 years, with a median of 53.50 years (IQR = 38). For women, the individual age range was 25 to 77 years, with a median of 37.50 years (IQR = 22). The age difference between the two genders was not statistically significant according to the Mann–Whitney U test (*p* = 0.639) (Figure 1b).

Clinical data for both the entire group of patients and by gender are summarized in Table 1. Statistical comparisons between the indicators of male and female patients were made according to the type and distribution of data.

The independent-samples *t*-test was used to draw a comparison between continuous variables and normal distribution, and in the absence of normal distribution, the Mann–Whitney U test was performed. For binary variables (1-present, 0–absent), the Fisher’s exact test was used.

Body mass index (BMI) had a mean value of 23.93 ± 3.50 for the whole group and presented similar values in men (23.07 ± 3.60) and women (24.65 ± 3.34) without significant difference (*p* = 0.191). Median GGT showed a significantly higher value in men at 51.00 (IQR = 34.77) compared with women at 26.00 (IQR = 29.25 (*p* = 0.045)).

No significant differences were found between male and female patients regarding the values of total bilirubin (*p* = 0.193), direct bilirubin (*p* = 0.210), ALP (*p* = 0.135), ASAT (*p* = 0.251) and ALAT (*p* = 0.283), the transverse section of the lesion (*p* = 0.694), and the longitudinal section of the lesion (*p* = 0.750).

In the entire group, 46.90% (n = 15) of patients reported regular alcohol consumption, with a significantly higher proportion in men (85.70%) compared with women (16.70%), *p* < 0.001. Smoking was observed in 37.50% (n = 12) of all patients, with a significantly higher proportion of male smokers (71.40%) compared with female smokers (11.10%), *p* = 0.001.

#### 4.1.2. SWV and Depth of the Region of Interest Values for pSWE and 2D-SWE in Patients with Simple Liver Cysts

Ten valid measurements of shear wave velocity (SWV) and depth of the region of interest were made on each simple liver cyst. The SWV values for pSWE m/s and 2D-SWE SWV m/s as well as the depth of the region of interest for pSWE in mm and 2D-SWE in mm, which are included in the present analysis, were the arithmetic mean values from all ten valid measurements (Table 2). Two of the variables (SWV for pSWE m/s and 2D-SWE m/s) showed a lack of normal distribution, and the average trend was represented by the median and interquartile range (IQR). Values for both genders were compared using the Mann–Whitney U test. The other two variables, including the depth of the region of interest for pSWE mm and for 2D-SWE mm, were found to be normally distributed, and the average trend was expressed by the arithmetic mean and standard deviation (SD). Genders were compared by the independent-samples *t*-test. The shear wave velocity (SWV) for pSWE had a median of 1.14 m/s (IQR = 0.27) in the whole group, 1.15 m/s (IQR = 1.13) in male patients, and 1.14 m/s (IQR = 0.23) in female patients, without a significant difference (*p* = 0.613). The depth of the region of interest for pSWE was 46.28 ± 7.10 mm in the whole group, 48.51 ± 7.50 mm in males, and 44.55 ± 6.44 mm in females, without a significant difference (*p* = 0.119). SWV for 2D-SWE showed a median of 1.00 m/s (IQR = 0.19) in the whole group, 0.97 m/s (IQR = 0.21) in male patients, and 1.00 m/s (IQR = 0.25) in female patients, with no significant difference between genders (*p* = 0.837). The depth of the region of interest for 2D-SWE had a mean value of 46.09 ± 6.07 mm in the study group, 47.61 ± 6.37 mm in males, and 43.65 ± 8.73 mm in females, with no significant difference (*p* = 0.218).

**Figure 2:**
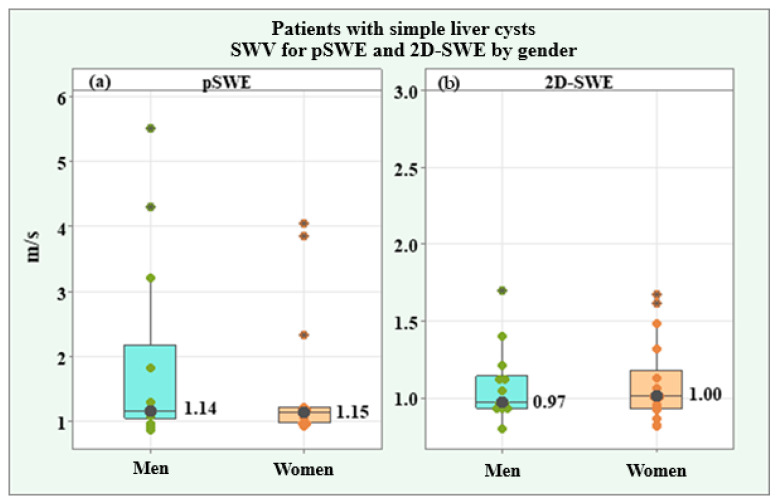
Box plots of SWV for pSWE (**a**) and SWV for 2D-SWE velocity (**b**) in male and female patients with simple liver cysts.

Figure 2 illustrates the similarities between the SWV values for pSWE and 2D-SWE in male and female patients through box plots of the medians, individual values, and interquartile ranges.

**Figure 3:**
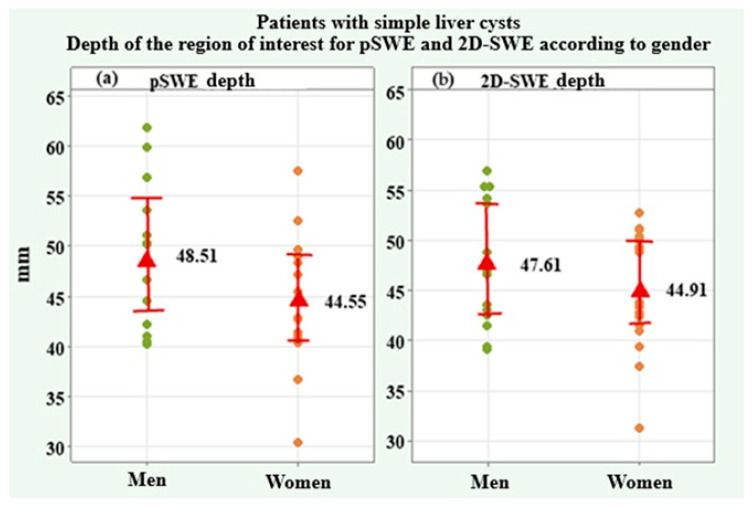
Plots of the depth of the region of interest in mm for pSWE (**a**) and 2D-SWE (**b**) in male and female patients.

Figure 3 presents graphs of the depth of the region of interest for pSWE and 2D-SWE in mm for male and female patients. Individual and mean values and 95% confidence intervals of SWV for pSWE and 2D-SWE are illustrated.

#### 4.1.3. SWV Reference Intervals for pSWE in Patients with Simple Liver Cysts

The lack of significant differences between male and female patients regarding SWV in pSWE and 2D-SWE justified the determination of reference intervals for the diagnosis of patients with simple liver cysts without distinguishing between genders. For this purpose, the specialized medical statistics program MedCalc version 22.021, 2024 was used.

The distribution of SWV values for pSWE in patients with simple liver cysts showed a lack of normal distribution and positive asymmetry (coefficient skewness = 1.62, *p* < 0.001). In such a case, and when there are no individual data with negative or zero values, transformation using a logarithmic function [x’ = log10(x), where x = the original dimension for each patient], is recommended to achieve a normal distribution. The MedCalc program version 22.021 (2024) was able to perform this function, after which the results were back-transformed (10x’) to the original scale. The reference intervals were determined using the Robust method, which is recommended for samples of less than 100 cases. The results are summarized in Table 3.

The lower reference limit was determined to be 0.35 m/s with a 90% confidence interval ranging from 0.26 m/s to 0.52 m/s. The upper reference limit was 3.33 m/s, with a 90% confidence interval ranging from 2.28 m/s to 4.61 m/s.

**Figure 4:**
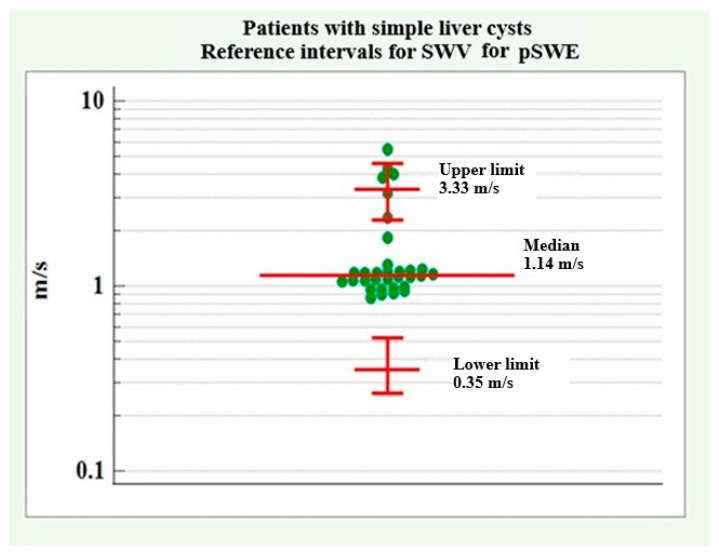
SWV reference intervals for pSWE velocity in patients with simple liver cysts.

Figure 4 presents a graph of the lower and upper reference limits of the shear wave velocity (SWV) for pSWE in patients with simple liver cysts.

#### 4.1.4. SWV Reference Intervals for 2D-SWE in Patients with Simple Liver Cysts

A lack of normal distribution was found in the distribution of SWV values for 2D-SWE in patients with simple liver cysts (coefficient skewness = 1.36, *p* < 0.01). Similar to pSWE, to achieve a normal distribution, the data were log-transformed and back-transformed to the original scale. The results are summarized in Table 4. The lower reference limit showed a value of 0.69 m/s with a 90% confidence interval of 0.62 m/s to 0.76 m/s. The upper reference limit was determined to be 2.05 m/s with a 90% confidence interval of 1.84 m/s to 2.29 m/s.

**Figure 5:**
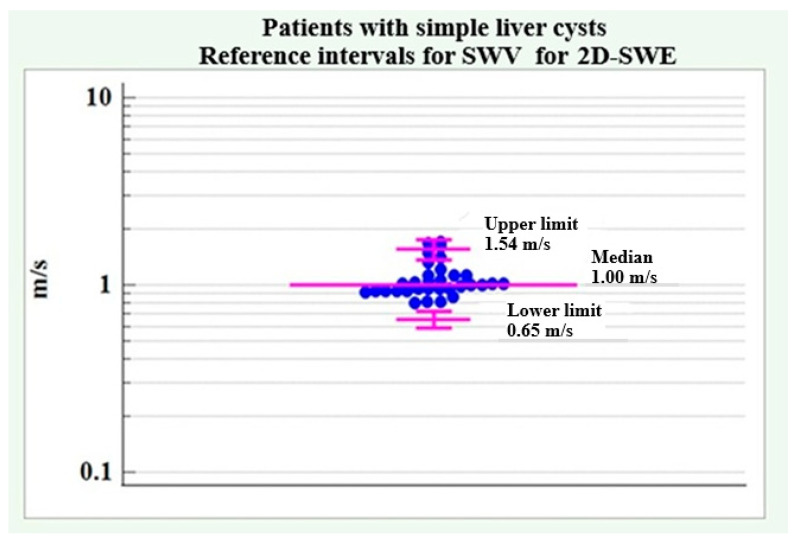
SWV reference intervals for 2D-SWE in patients with simple liver cysts.

Figure 5 illustrates the reference limits of SWV for 2D-SWE in patients with simple liver cysts.

#### 4.1.5. Summary of the Main Trends Regarding Patients with Simple Liver Cysts

1. Demographic and clinical data for patients with simple liver cysts showed significant differences between men and women regarding:
  - GG with significantly higher values in men compared with women.
  - Alcohol use and smoking, with a significantly higher relative proportion in male patients. The results are summarized in Table 5.
2. No significant difference was found between the two genders regarding the SWV values for pSWE and 2D-SWE in patients with simple liver cysts.
3. The following reference ranges for SWV for pSWE and 2D-SWE were determined for patients with simple liver cysts. The results are summarized in Table 6.

### 4.2. Information on Patients with Liver Hemangiomas

#### 4.2.1. Background Information on Patients with Liver Hemangiomas

**Figure 6:**
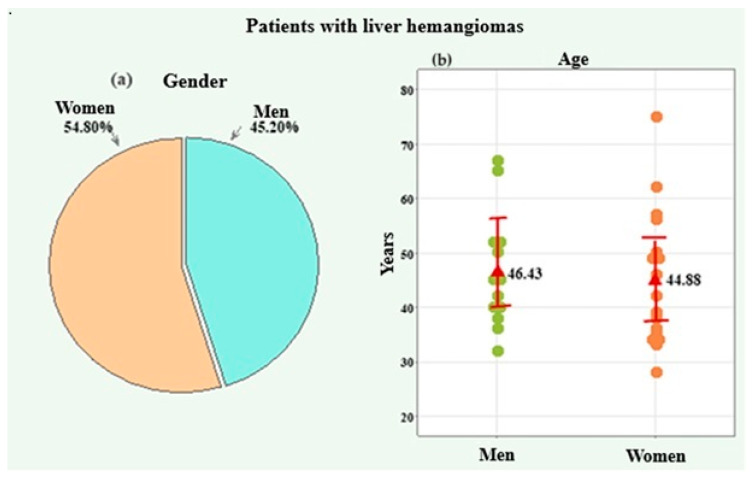
Distribution by gender (**a**) and age (**b**) of the patients with liver hemangiomas.

The liver hemangiomas group included 31 patients, of whom 14 (45.20%) were male and 17 (54.80%) were female (Figure 6a). The age of the patients was presented by the arithmetic mean and standard deviation (SD). The age of the participants regardless of gender ranged from 28 to 75 years, with a mean of 45.58 ± 11.28 years. For men, the age range was from 32 to 67 years, with a mean age of 46.43 ± 10.14 years. For women, the individual age ranged from 28 to 75 years, with a mean age of 44.88 ± 12.40 years.

The age difference between the two genders was not statistically significant according to the *t*-test comparison (*p* = 0.711) (Figure 6b).

Clinical data for both the entire group of patients and subdivisions by gender are summarized in Table 7. Statistical comparisons between the indicators of male and female patients were made according to the type and distribution of data. For a comparison of continuous variables with normal distribution, an independent-samples *t*-test was used, and in the absence of normal distribution, the Mann–Whitney U test was performed. For binary variables (1—yes, 0—no), Fisher’s exact test was applied. Body mass index (BMI) had a median of 22.90 (IQR = 3.80) for the entire group and similar values for men (23.45, IQR = 5.20) and women (22.80, IQR = 4.00), without a significant difference (*p* = 0.246).

No significant differences were found between male and female patients regarding the values of GGT (*p* = 0.769), total bilirubin (*p* = 0.234), direct bilirubin (*p* = 0.834), ALP (*p* = 0.377), ALAT (*p* = 0.769), the size of the cross-sectional area of the lesion (*p* = 0.769), and the longitudinal area of the lesion (*p* = 0.252). The only significant gender difference was found for ASAT, with higher values in men 29.50 (IQR = 10.50) compared with women 21.00 (IQR = 10.00), *p* = 0. 044. Of all patients, 45.20% (n = 14) reported alcohol consumption, with a significantly higher proportion of male smokers (71.40%) compared with female smokers (23.50%), *p* = 0.012. Smoking was also reported by 45.20% (n = 14) of all participants, 50.00% of men and 41.20% of women, without a significant difference (*p* = 0.725).

#### 4.2.2. SWV and Depth of the Region of Interest Values for pSWE and 2D-SWE in Patients with Liver Hemangiomas

Ten valid measurements of shear wave velocity (SWV) and depth of interest were made for each liver hemangioma. The SWV values for pSWE m/s and 2D-SWE m/s as well as the depth of the region of interest for pSWE mm and 2D-SWE mm, all of which were included in the present analysis, were the arithmetic mean values of all ten dimensions (Table 8). Two of the dimensions, SWV for pSWE m/s and 2D-SWE m/s, showed a lack of normal distribution, and the average trend was represented by the median and interquartile range (IQR). The values for both genders were compared using the Mann–Whitney U test. On the other hand, the depth of the region of interest for pSWE mm and 2-D-SWE mm was found to be normally distributed, and the average trend was expressed by the arithmetic mean and standard deviation (SD). The genders were compared using the independent-samples *t*-test. The shear wave velocity (SWV) for pSWE had a median of 1.36 m/s (IQR = 0.74) for the group as a whole, 1.38 m/s (IQR = 0.72) in male patients, and 1.36 m/s (IQR = 0.81) in female patients, without a significant difference (*p* = 0.544). The depth of the region of interest for pSWE was 44.89 ± 9.26 mm in the whole group, 45.24 ± 10.52 mm in males, and 44.59 ± 8.40 mm in females, without a significant difference (*p* = 0.850). SWV for 2D-SWE had a median of 1.34 m/s (IQR = 0.19) in the whole group, 1.38 m/s (IQR = 0.17) in male patients, and 1.32 m/s (IQR = 0.46) in female patients, with no significant difference between genders (*p* = 0.653). The depth of the region of interest for 2D-SWE had a mean value of 44.58 ± 7.93 mm in the study group, respectively 46.56 ± 7.80 mm in males and 42.95 ± 7.71 mm in females, with no significant difference (*p* = 0.208).

**Figure 7:**
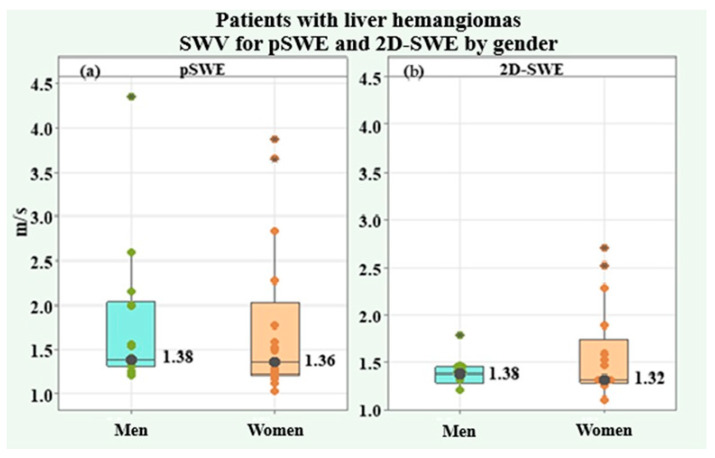
Box plots of SWV for pSWE (**a**) and SWV for 2D-SWE (**b**) in male and female patients with liver hemangiomas.

Figure 7 illustrates the similar SWVs for pSWE and 2D-SWE, in male and female patients, by box plots of the medians, individual values, and interquartile ranges.

**Figure 8:**
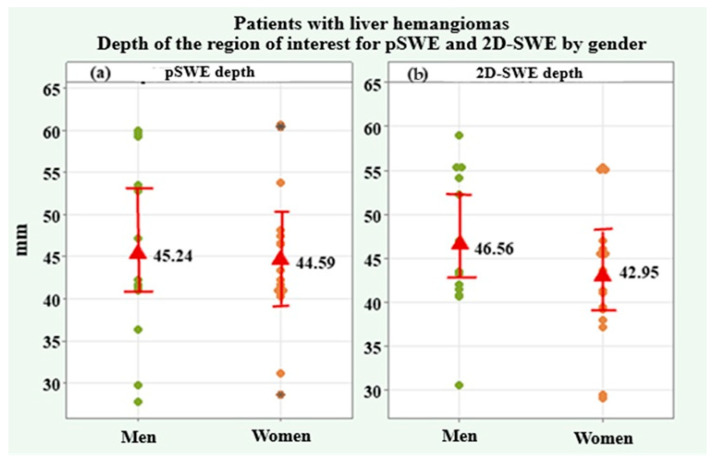
Plots of the depth of the region of interest in mm for pSWE (**a**) and 2D-SWE (**b**) in male and female patients with liver hemangiomas.

Figure 8 presents the depth of the region of interest for pSWE and 2D-SWE in mm in the male and female patients. Individual and mean values and 95% confidence intervals are illustrated.

#### 4.2.3. Reference Intervals of SWV for pSWE in Patients with Liver Hemangiomas

Reference intervals for the diagnosis of patients with liver hemangiomas were established without distinguishing between genders due to the lack of a significant difference between male and female patients regarding SWV for pSWE. The distribution of SWV values for pSWE in patients with liver hemangiomas showed a lack of normal distribution and positive asymmetry (coefficient skewness = 1.35, *p* < 0.001). To achieve a normal distribution, the data were logarithmically transformed [x’ = log10(x), where x = the original dimension for each patient], and then the results were back-transformed (10x’) to the original scale. Reference intervals were determined using the Robust method, which is recommended for samples of less than 100 cases.

The lower reference limit was determined to be 0.45 m/s with a 90% confidence interval covering the range 0.38 m/s to 0.56 m/s. The upper reference limit showed a value of 3.06 m/s with a 90% confidence interval of 2.49 m/s to 3.06 m/s.

**Figure 9:**
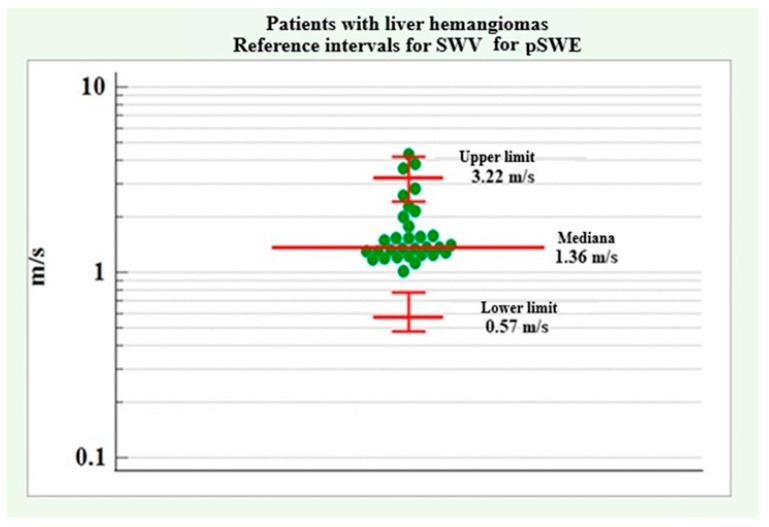
SWV reference intervals for pSWE in patients with liver hemangiomas.

Figure 9 presents a graph of the lower and upper reference limits of SWV for pSWE in patients with liver hemangiomas.

#### 4.2.4. SWV Reference Intervals for 2D-SWE in Patients with Liver Hemangiomas

SWV reference intervals for 2D-SWE applied to patients with liver hemangiomas without distinguishing between genders, as no significant difference was found between male and female patients regarding SWV for 2D-SWE.

Data were logarithmically transformed and back-transformed to the original scale due to a lack of normal distribution of SWV for 2D-SWE (coefficient skewness = 1.54, *p* < 0.001). The lower reference limit showed a value of 0.80 m/s with a 90% confidence interval of 0.70 m/s to 0.96 m/s. The upper reference limit was determined at 2.27 m/s with a 90% confidence interval of 1.90 m/s to 2.61 m/s.

**Figure 10:**
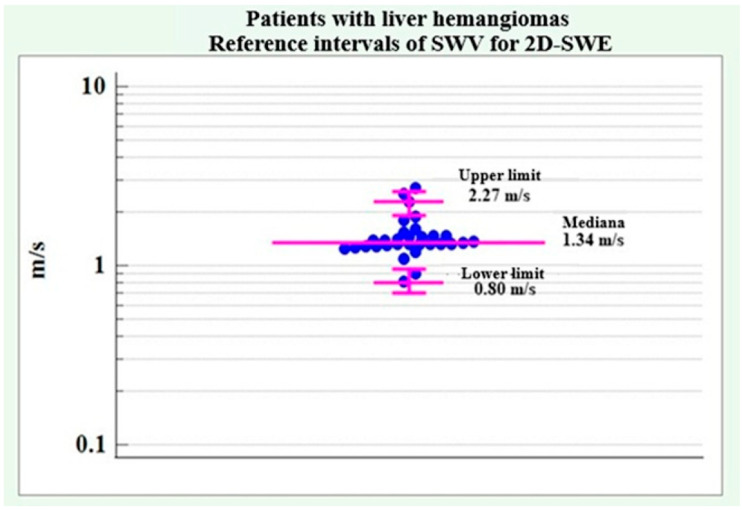
SWV reference intervals for 2D-SWE in patients with liver hemangiomas.

Figure 10 illustrates the reference limits of SWV for 2D-SWE in patients with liver hemangiomas.

#### 4.2.5. Summary of Main Trends Regarding Patients with Liver Hemangiomas

1. Demographic and clinical data for patients with liver hemangiomas did not show any significant differences between men and women, except for alcohol consumption, which showed a significantly higher relative proportion in male patients. The results are summarized in Table 9.
2. No significant difference was found between the two genders regarding SWV values for pSWE and 2D-SWE in patients with liver hemangiomas.
3. The following reference ranges of SWV for pSWE and 2D-SWE were determined in patients with liver hemangiomas. The results are summarized in Table 10.

## 5. Discussion

The focal liver lesions examined in this task were simple liver cysts and liver hemangiomas. These represent the most common benign focal liver lesions encountered daily in clinical practice during routine abdominal ultrasonography. The lack of information in the literature worldwide regarding the elastographic stiffness of simple hepatic cysts was the main reason for their inclusion in our study.

The ultrasonographic appearance of liver hemangiomas and their differential diagnosis from hepatic neoplasia often necessitates contrast-enhanced imaging. The need for a non-invasive, harmless, easily accessible, and highly informative method has directed much attention toward investigating the potential of pSWE and 2D-SWE in differentiating focal liver lesions.

The study included 32 patients with simple hepatic cysts and 31 patients with liver hemangiomas, all confirmed by contrast-enhanced computed tomography. Ten valid measurements were taken to establish reference ranges for shear wave velocity (SWV) using pSWE and 2D-SWE in those patients.

The pSWE SWV values (median) for simple hepatic cysts showed an average trend of 1.14 m/s, with an upper limit of 3.33 m/s and a lower limit of 0.35 m/s. For 2D-SWE, the average trend was 1.00 m/s, with an upper limit of 1.54 m/s and a lower limit of 0.65 m/s.

To date, no similar analysis has been reported in the available literature. Due to the heterogeneous results obtained with both elastographic methods, further research on larger patient groups is necessary to validate the results before discussing their clinical application.

Demographic and clinical data for patients with simple hepatic cysts revealed significantly higher gamma-glutamyl transferase (GGT) levels in men compared with women as well as a higher prevalence of alcohol consumption and smoking among males. No significant gender-related differences were observed in SWV values for either pSWE or 2D-SWE.

For liver hemangiomas, the average trend in pSWE was 1.36 m/s, with an upper limit of 3.22 m/s and a lower limit of 0.57 m/s. For 2D-SWE, the average trend was 1.34 m/s, with an upper limit of 2.27 m/s and a lower limit of 0.80 m/s. Similar results were obtained in the study by Cho et al. [25], where the pSWE SWV for liver hemangiomas was 1.51 ± 0.71 m/s, and in the study by Guo et al. [26], where it was 1.48 ± 0.70 m/s.

In a study conducted by Heide et al. [27], the results for patients with liver hemangiomas were considerably higher (2.36 m/s) compared with our data. This variability in values has been explained based on the number of fibrous septa dividing the dilated vascular spaces [25]. Hemangiomas are composed of numerous vascular channels filled with blood, which is why they were not expected to be stiff, and the obtained results tended to be lower. In contrast, hemangiomas with pathological inclusions such as calcifications, sclerosis, or vascular thrombosis would be stiffer and have higher stiffness values [28]. Since these studies analyzed a relatively small number of patients with hemangiomas, this discrepancy should be investigated further by including a larger number of cases.

The SWV results obtained using 2D-SWE in previous studies are expressed in kPa rather than m/s. Additional studies on larger patient groups with results presented in m/s are needed before discussing the clinical applicability of this method.

Demographic and clinical data for patients with liver hemangiomas did not show significant gender-related differences, except for a notably higher prevalence of alcohol consumption among male patients.

## 6. Conclusions

The accurate diagnosis of liver diseases is of paramount importance for the approach and treatment of individual benign hepatic lesions. With the advances in medicine over recent decades, non-invasive methods for evaluating the hepatic parenchyma have become an indispensable part of routine clinical practice. These methods offer a fundamentally new perspective on assessing pathologically altered tissues based on changes in their elasticity that is determined by the degree of deformation in response to an external force applied onto them.

Elastographic methods can help predict the nature of focal liver lesions, potentially avoiding more invasive procedures like liver biopsy or imaging techniques such as CT and MRI. Additionally, elastography plays a key role in monitoring the progression of liver fibrosis and assessing the treatment response in patients with chronic liver diseases.

Overall, the need for innovative methods that are more informative, but easily accessible and applicable in everyday clinical practice, has given rise to the idea of exploring elastographic techniques such as pSWE and 2D-SWE as diagnostic tools for focal liver lesions [29,30]. Therefore, the validation of these findings calls for further studies and exploration in this direction.

## Figures and Tables

**Table 1:** Data of patients with simple liver cysts.

Values	Total (*n* = 63)	Male (*n* = 28)	Female (*n* = 35)	***p***
BMI o mean (SD)	23.93 (3.50)	23.07 (3.60)	24.65 (3.34)	0.191 ^t^
GGT o median (IQR)	38.00 (35.50)	51.00 (34.77)	26.00 (29.25)	0.045 ^U^
Total bilirubin o median (IQR)	15.05 (6.10)	17.45 (8.20)	14.45 (5.27)	0.193 ^U^
Direct bilirubin o mean (SD)	5.40 (2.15)	5.95 (2.59)	4.97 (1.68)	0.210 ^t^
ALP o median (IQR)	58.00 (38.75)	57.00 (26.75)	73.50 (38.00)	0.135 ^U^
ASAT o median (IQR)	21.00 (14.75)	19.50 (13.25)	22.00 (15.00)	0.251 ^U^
ALAT o median (IQR)	22.50 (18.50)	27.50 (22.50)	20.00 (15.50)	0.283 ^U^
Cross-sectional area of lesion (mm) o median (IQR)	29.00 (16.25)	29.00 (25.25)	30.00 (12.00)	0.694 ^U^
Longitudinal section of lesion (mm) o median (IQR)	31.50 (14.50)	32.38 (12.40)	33.62 (11.63)	0.750 ^U^
Alcohol n (%)	15 (46.90%)	12 (85.70%)	3 (16.70%)	<0.001 ^f^
Smoking n (%)	12 (37.50%)	10 (71.40%)	2 (11.10%)	0.001 ^f^

IQR—interquartile range, GGT—gamma-glutamyltransferase; ALP—alkaline phosphatase; ASAT—aspartate aminotransferase; ALAT—alanine aminotrasferase; ^t^ Test for independent samples; ^U^ Mann–Whitney U test; ^f^ Fisher’s exact test.

**Table 2:** SWV values and depth of the region of interest for pSWE and 2D-SWE in patients with simple liver cysts.

Values	Total (*n* = 63)	Male (*n* = 28)	Female (*n* = 35)	*p*
pSWE SWV (m/s) o median (IQR) o minimum–maximum	1.14 (0.27) 0.86–5.51	1.15 (1.13) 0.86–5.51	1.14 (0.23) 0.92–4.04	0.613 ^U^
pSWE depth (mm) o mean (SD) o minimum–maximum	46.28 (7.10) 30.34–61.86	48.51 (7.50) 40.14–61.86	44.55 (6.44) 30.34–57.57	0.119 ^t^
2D-SWE SWV (m/s) o median (IQR) o minimum–maximum	1.00 (0.19) 0.80–1.70	0.97 (0.21) 0.80–1.70	1.00 (0.25) 0.81–1.67	0.837 ^U^
2-D-SWE depth (mm) o mean (SD) o minimum–maximum	46.09 (6.07) 31.30–56.90	47.61 (6.37) 39.10–56.90	44.91 (5.73) 31.30–52.70	0.218 ^t^

IQR—interquartile range, pSWE—point shear wave elastography; 2D-SWE—two-dimensional shear wave elastography; ^t^ *t*-test for independent samples; ^U^ Mann–Whitney U test.

**Table 3:** Results of the analysis for determining the reference intervals for SWV of pSWE in patients with simple liver cysts.

Back-Transformed After Logarithmic Transformation	
Number of cases	*n* = 32
Lower reference limit	0.86
Upper reference limit	5.51
Mean	1.38
Median	1.14
Shapiro–Wilk test	*p* = 0.11
Robust method	
Lower limit 90% Confidence interval (90% CI)	0.35 (0.26–0.52)
Upper limit 90% Confidence interval (90% CI)	3.33 (2.28–4.61)

**Table 4:** Results of the analysis to determine reference intervals for 2D-SWE in patients with simple liver cysts.

Back-Transformed After Logarithmic Transformation	
Number of cases	n = 32
Lower reference limit	0.80
Upper reference limit	1.70
Mean	1.06
Median	1.00
Shapiro–Wilk test	*p* = 0.32
Robust method	
Lower limit 95% Confidence interval (95% CI)	0.65 (0.59–0.72)
Upper limit 95% Confidence interval (95% CI)	1.54 (1.35–1.75)

**Table 5:** Gender differences.

	Male	Female
Alcohol	85.70%	16.70%
Smoking	71.40%	11.10%

**Table 6:** Reference ranges.

SWV	Median	Upper Limit	Lower Limit
pSWE	1.14 m/s	3.33 m/s	0.35 m/s
2D-SWE	1.00 m/s	1.54 m/s	0.65 m/s

**Table 7:** Data of patients with liver hemangiomas.

Values	Total (*n* = 63)	Male (*n* = 28)	Female (*n* = 35)	*p*
BMI o median (IQR)	22.90 (3.80)	23.45 (5.20)	22.80 (4.00)	0.246 ^U^
GGT o median (IQR)	23.00 (15.00)	23.50 (16.25)	22.00 (15.50)	0.769 ^U^
Total bilirubin o mean (SD)	13.82 (3.08)	13.08 (2.85)	14.42 (3.22)	0.234 ^t^
Direct bilirubin o mean (SD)	4.20 (1.52)	4.27 (1.48)	4.15 (1.59)	0.834 ^t^
ALP o median (IQR)	77.00 (23.00)	76.00 (34.00)	83.00 (18.50)	0.377 ^U^
ASAT o median (IQR)	25.00 (13.00)	29.50 (10.50)	21.00 (10.00)	0.044 ^U^
ALAT o median (IQR)	22.00 (15.00)	21.50 (11.00)	23.00 (17.00)	0.769 ^U^
Cross-sectional area of lesion (mm) o median (IQR)	31.43 (12.56)	29.93 (10.23)	31.24 (12.54)	0.756 ^t^
Longitudinal section of lesion (mm) o mean (SD)	33.07 (11.90)	30.55 (12.29)	36.04 (13.59)	0.252 ^t^
Alcohol n (%)	14 (45.20%)	10 (71.40%)	4 (23.50%)	0.012 ^f^
Smoking n (%)	14 (45.20%)	7 (50.00%)	7 (41.20%)	0.725 ^f^

IQR—interquartile range, GGT—gamma-glutamyltransferase; ALP—alkaline phosphatase; ASAT—aspartate aminotransferase; ALAT—alanine aminotrasferase; ^t^ Test for independent samples; ^U^ Mann–Whitney U test; ^f^ Fisher’s exact test.

**Table 8:** SWV values and depth of the region of interest for pSWE and 2D-SWE in patients with liver hemangiomas and by gender.

Values	Total (*n* = 63)	Male (*n* = 28)	Female (*n* = 35)	*p*
pSWE SWV (m/s) o median (IQR) o minimum–maximum	1.36 (0.74) 1.02–4.35	1.38 (0.72) 1.21–4.35	1.36 (0.81) 1.02–3.87	0.544 ^U^
pSWE depth (mm) o mean (SD) o minimum–maximum	44.89 (9.26) 27.80–60.60	45.24 (10.52) 27.80–59.96	44.59 (8.40) 28.52–60.60	0.850 ^t^
2D-SWE SWV (m/s) o median (IQR) o minimum–maximum	1.34 (0.19) 0.82–2.70	1.38 (0.17) 0.82–1.79	1.32 (0.46) 1.10–2.70	0.653 ^U^
2-D-SWE depth (mm) o mean (SD) o minimum–maximum	44.58 (7.93) 29.00–59.00	46.56 (7.80) 30.50–59.00	42.95 (7.71) 29.00–55.30	0.208 ^t^

IQR—interquartile range, pSWE—point shear wave elastography; 2D-SWE—two-dimensional shear wave elastography; ^t^ *t*-test for independent samples; ^U^ Mann–Whitney U test.

**Table 9:** Gender differences.

	Male	Female
Alcohol	71.40%	23.50%

**Table 10:** Reference ranges.

SWV	Median	Upper Limit	Lower Limit
pSWE	1.36 m/s	3.22 m/s	0.57 m/s
2D-SWE	1.34 m/s	2.27 m/s	0.80 m/s

## Data Availability

The original contributions presented in this study are included in the article. Further inquiries can be directed to the corresponding authors. The authors have reviewed and edited the output and take full responsibility for the content of this publication.

## Abbreviations

The following abbreviations are used in this manuscript:

pSWE	Point shear wave elastography
2D-SWE	Two-dimensional shear wave elastography
